# Solid Truss to Shell Numerical Homogenization of Prefabricated Composite Slabs

**DOI:** 10.3390/ma14154120

**Published:** 2021-07-23

**Authors:** Natalia Staszak, Tomasz Garbowski, Anna Szymczak-Graczyk

**Affiliations:** 1 Research and Development Department, FEMat Sp. z o. o., Romana Maya 1, 61-371 Poznań, Poland; natalia.staszak@fematproject.pl; 2 Department of Biosystems Engineering, Poznan University of Life Sciences, Wojska Polskiego 50, 60-627 Poznań, Poland; tomasz.garbowski@up.poznan.pl; 3 Department of Construction and Geoengineering, Poznan University of Life Sciences, Piątkowska 94 E, 60-649 Poznań, Poland

**Keywords:** numerical homogenization, prefabricated floor slab, concrete, composite structure, strain energy equivalence

## Abstract

The need for quick and easy deflection calculations of various prefabricated slabs causes simplified procedures and numerical tools to be used more often. Modelling of full 3D finite element (FE) geometry of such plates is not only uneconomical but often requires the use of complex software and advanced numerical knowledge. Therefore, numerical homogenization is an excellent tool, which can be easily employed to simplify a model, especially when accurate modelling is not necessary. Homogenization allows for simplifying a computational model and replacing a complicated composite structure with a homogeneous plate. Here, a numerical homogenization method based on strain energy equivalence is derived. Based on the method proposed, the structure of the prefabricated concrete slabs reinforced with steel spatial trusses is homogenized to a single plate element with an effective stiffness. There is a complete equivalence between the full 3D FE model built with solid elements combined with truss structural elements and the simplified homogenized plate FE model. The method allows for the correct homogenization of any complex composite structures made of both solid and structural elements, without the need to perform advanced numerical analyses. The only requirement is a correctly formulated stiffness matrix of a representative volume element (RVE) and appropriate formulation of the transformation between kinematic constrains on the RVE boundary and generalized strains.

## 1. Introduction

Prefabricated concrete structures such as floor slabs, girders, and columns have numerous advantages, including (a) quality standard, (b) saving of formwork, (c) short construction time, (d) durability of the structure, and (e) very low energy consumption [1,2,3]. That is why prefabricated concrete elements, and in particular composite structures, have been widely used in industrial and residential buildings all over the world in recent decades [2]. Floor slabs and girders are one of the most frequently used prefabricated structural elements among all prefabricated structures [4].

One of the most popular prefabricated composite floor-slab is the so-called “Filigree” ceiling, which is used all over Europe. It is a typical example of a semi-prefabricated ceiling used in industrial, residential, rural, and general construction, and it allows architects to follow their creativity, which this type of ceiling does not limit. In Poland, this ceiling first appeared in 1996, while in Europe these structures could be found already in 1964–1965. The most important feature in this type of floor is that the strength of floor slabs is adjusted to a specific load, in accordance with conditions prevailing during the use of the floor.

Over the past years, various numerical studies have been developed to better understand the mechanical behaviour of precast composite structures. To present the global behaviour of prefabricated structures, various models have been developed. From the macro level, where fibre elements are used to simulate prefabricated beams, slabs, columns [5,6,7], and sandwich layered shells are used to simulate prefabricated slabs. Tzaros et al. [8] studied the bending behaviour of composite slabs. Modelling of structural elements at the macro level, however, presents some obstacles to, for example, an accurate simulation of the contact between a precast element and concrete poured in place. In the work published by Abdullah and Easterling [9], a force balance method was developed to calculate the shear-slip relationship in FEM modelling of composite slabs. Ren et al. [10] proposed a full 3D FE model of precast concrete bridge panels to model its nonlinear behaviour using a concrete damage plasticity constitutive model. The complete procedure for concrete damage plasticity model calibration is given in the work by Jankowiak and Łodygowski [11]. Other material models for concrete can be used for numerical analysis provided their constitutive parameters are properly identified [12,13,14].

A three-dimensional (3D) solid FE model was also used in the work of Gholamhoseini et al. [15], where interface elements were introduced to present the bonding between steel and concrete. Nonlinear 3D FEM models of composite panels were also used to present slip mechanics in “pull-out” tests [16]. On the other hand, the effective FEM model used to recreate the longitudinal shear behaviour of composite slabs with a profiled sheet was presented in the work by Ríos et al. [17]. In general, with the development of computational speed, 3D finite element modelling has become a promising tool for the numerical analysis of prefabricated concrete structures, primarily due to its ability to describe complex relationship between composite elements.

If complex analysis is not particularly required, time consuming modelling of full 3D FE models is not economical. In such cases, the complex multi-layer composite cross-section can be replaced by a single-layer model with equivalent properties, which ensures that its behaviour is very close to the reference model. This kind of simplification is known as homogenization. Numerous studies have dealt with the homogenization of complex sections for dozens of years. In 2003, Hohe presented homogenization methods based on strain energy [18]. The author applied a procedure in the context of homogenization of sandwich panels with a honeycomb core. It uses strain energy with the assumption of mechanical equivalence between a simplified model and the representative volume element (RVE) of a sample. Another type of homogenization is a periodic homogenization method proposed by Buanic et al. [19]. It allows for calculating both the equivalent shearing and bending characteristics of the periodic plates and membrane. Biancolini used the strain energy equivalence between a single layer equivalent model and the numerical model of representative volume element (RVE) to compute the bending properties of corrugated core panels and membrane [20]. This method was later updated by Garbowski and Gajewski [21]. In the work by Marek and Garbowski [22], the compilation and comparison of various methods to homogenization of composite plates with corrugated and sandwich core were presented. A slightly different approach, based on inverse analysing can be found in the study by Garbowski and Marek [23]. An interesting approach to periodic composites based on the numerical implantation of asymptotic homogenization method is presented in [24,25,26]. The textile composite, on the other hand, can be homogenized by a multiscale approach based on mechanics of a structure genome [27,28,29]. The discussion about the differences between the static and dynamic homogenization can be found in the wark by Tallarico et al. [30]

This paper presents an extension of the homogenization method presented by Biancolini [20] and later by Garbowski and Gajewski [21]. Originally, this method was developed for homogenization of shell structures. In this paper, the extension of the method is presented so that it can be applied to structures made of both solid and structural components and allows the transverse shear to be taken into account during homogenization. The transverse shear significantly affects the mechanical behaviour of, e.g., sandwich structures; therefore, this issue has been addressed by many researchers, e.g., [31,32]. A similar effect is expected in the case of a structure consisting of a concrete slab with spatial truss reinforcement. The proposed approach uses the principles of strain energy equivalence between the simplified single layer shell model and the full 3D finite element RVE model of the prefabricated composite slab. The results obtained by the proposed homogenization method were compared with the ones obtained through the full numerical analysis of 3D modelling. The calculated displacements using the full 3D FEM model and the simplified shell (equivalent) model are in very good agreement.

## 2. Materials and Methods

### 2.1. Precasted Composite Slabs

The work presents a solid truss to shell homogenization method using as an example a Filigree precast slab. Therefore, the subsection below provides some basic information about the ceiling. The Filigree plate is a prefabricated composite floor consisting of a reinforced concrete slab, spatial trusses, and concrete topping laid on the construction site. The thickness of prefabricated plates usually varies between 4.5 and 7 cm. The structural thickness of the ceiling is usually between 15 and 30 cm. Due to transport and optimal material consumption, filigree slabs are most often found in dimensions from 2.4 to 10 m (length) and 0.6 to 2.5 m (width). They can be formed in any shape from a trapezoid, rectangle, and triangle to a semicircle. Additionally, the location of the mounting holes can be taken into account already at the design stage. Slabs are made of concrete of at least C20/25 class, and their weight is on average 125–200 $\mathrm{kg}/m^{2}$.

Reinforcement of prefabricated slabs is calculated in the same way as for monolithic ceilings. The main reinforcement of prefabricated slabs formwork is longitudinal bars, while transverse reinforcement only plays a role of a distributed reinforcement. In addition, slabs are joined on the construction site with the use of nets, which helps to avoid the keying effect found in various foundations. Moreover, prefabricated slabs make also a permanent formwork.

Filigree slabs use the previously mentioned space trusses. They constitute an additional reinforcement of the ceiling and its stiffening. The trusses are arranged parallel to the longer side of the slab, with a spacing not exceeding 75 cm. We can distinguish, among others, D, E, EK, EQ, SWE, and JD types of trusses. Recommended bar diameters, spacing of bottom bars, and diagonals as well as other necessary information can be found in the National Technical Assessment [33].

**Figure 1:**
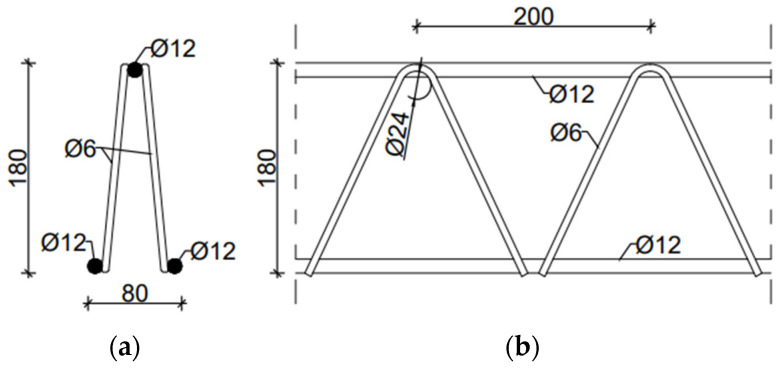
D-type spatial truss (**a**) X-X view; (**b**) Y-Y view.

To demonstrate the homogenization technique proposed, two types of trusses were utilized. These were D and EQ types of spatial trusses. They consisted of an upper flange in the form of a single steel bar, a lower flange in the form of two bars, and steel diagonals arranged in two planes. The D-type truss consisted of a truss strip made of bars with a diameter of 12 mm and cross-braces made of bars with a diameter of 6 mm, see Figure 1. Permissible bar diameters were as follows: belts—from 5 to 14 mm, crosses 5–7 mm. The truss height was assumed to be 18 cm, and the diagonal spacing was 20 cm.

**Figure 2:**
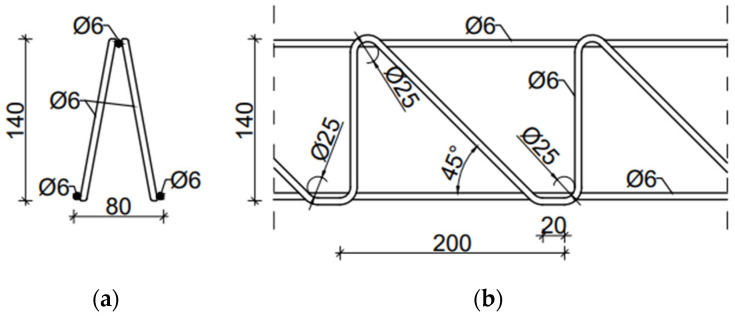
EQ-type spatial truss (**a**) X-X view; (**b**) Y-Y view.

The EQ-type spatial truss was made entirely of 6 mm diameter rods. The external spacing of lower bars was 80 mm, and the height and spacing of diagonals were 140 mm and 200 mm, respectively. Figure 2 shows the cross-section and dimensions of the EQ-type truss.

**Figure 3:**
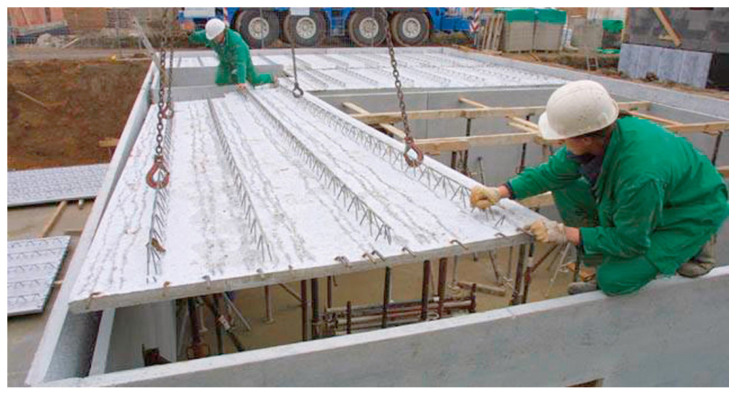
Filigree ceiling on the construction site.

Filigree ceilings are used in industrial as well as residential, general, and rural construction [34], see Figure 3. The entire panel production process, from the delivery of the architectural design to the delivery of panels to the construction site, does not take long. In the case of a single-family house, the production time is approximately 3–4 days, and prefabricated panels themselves are made in production plants on special heated tables.

**Figure 4:**
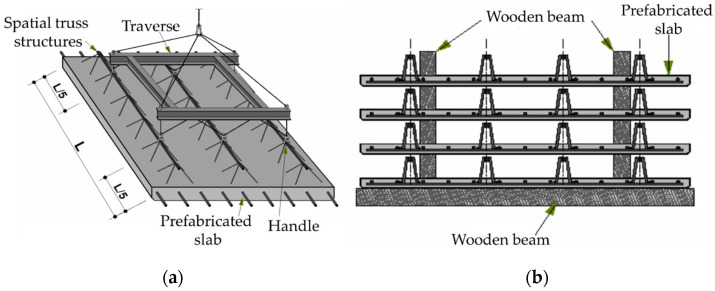
Filigree ceiling: (**a**) transport (assembly); (**b**) storage.

Prefabricated elements, before they fulfil their final task, go through many stages in which they are exposed to different support conditions and are subject to different loads. In the case of Filigree ceiling, transport and storage, as permanent formwork and as part of the structure phase, can be distinguished. During transport (assembly), a prefabricated slab is mainly affected by its own weight. The action of wind and snow can be neglected since the assembly of elements takes place in good weather conditions. In this situation, support is provided by the ones attached to spatial trusses [35], see Figure 4a.

The next phase is storage. Prefabricated slabs are placed on top of each other, separated by wooden beams [35], as shown in Figure 4b. When the slab has been embedded in the structure and concreted, it transfers the loads from the weight of the ceiling and other layers placed on it. In addition, it must be able to withstand possible variable loads, for example, from people, furniture, and partitions. Filigree ceilings are able to transfer a uniformly distributed load of 10 $\mathrm{kN}/m^{2}\;$ depending on the span and thickness of the board.

All the described special features of Filigree ceilings, as well as the multitude of load cases to which the panels may be subjected during transport and assembly make the need for quick calculations of panel deflections highly justified. This can be achieved in two ways: (i) using a complex model of the full 3D structure of the slab (reinforced slab and spatial truss) or (ii) using a simplified model (single-layer shell structure) with effective parameters allowing for quick and precise calculations of different panel variants. Both approaches use the finite element (FE) method. In the first one, both solid and truss elements and a full model (with several thousand FEs) of the complex geometry of the entire slab need to be applied, in the latter—a small representative model (RVE) should be constructed for proper homogenization so that during the next step a simple plate model (with several dozen FEs) could be used for calculation. In the following section, both numerical approaches are described.

### 2.2. Full 3D Numerical Model

**Figure 5:**
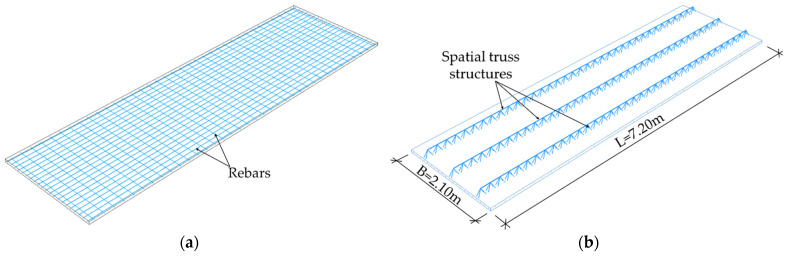
Filigree ceiling slab with D-type trusses (**a**) rebars; (**b**) concrete slab.

In order to compute the reference deflections (but also to validate the proposed homogenization method), two examples were considered. In both cases, FE commercial software (ABAQUS FEA [36]) was used. The results obtained from the full 3D models were further compared with the results obtained from the simplified shell model (after homogenization). In the first model, a Filigree ceiling slab with D-type trusses was modelled as a simply supported plate. The slab was modelled as 3D solid concrete structure. The slab was 2.10 m wide, 7.20 m long, and 0.06 m thick, see Figure 5b. The reinforcement of the slab and the trusses were modelled as steel wire structures. In this model, D-type trusses with the dimensions and cross-section shown in Figure 1 were used. The reinforcement mesh was modelled as the main reinforcement consisting of bars with diameter of ϕ = 10 mm, spaced every 100 mm, and the separating reinforcement with bars ϕ = 6 mm every 200 mm (shorter bars), see Figure 5a.

**Figure 6:**
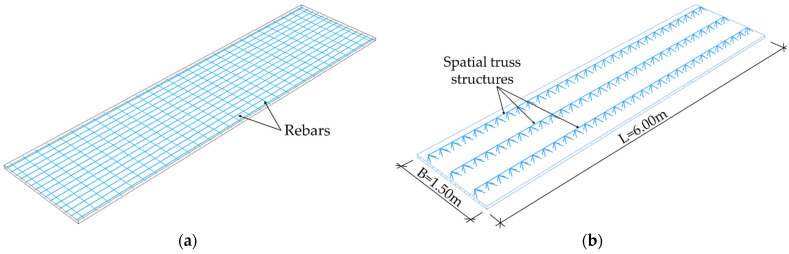
Filigree ceiling slab with EQ-type trusses (**a**) rebars; (**b**) concrete slab.

In the numerical model 2, a Filigree ceiling slab with EQ-type trusses was modelled. The dimension of EQ-type trusses is shown in Figure 2. The trusses and reinforcement were modelled as steel wire structures, whereas the slab was made of concrete and modelled as a 3D solid. Reinforcement of the slab had the same properties as in model 1, i.e., the main reinforcement was made of bars ϕ = 10 mm every 100 mm, and the separating reinforcement was made of bars with a diameter of ϕ = 6 mm every 200 mm. The slab to analyse was 1.50 m wide, 6.00 m long, and 0.05 m thick, see Figure 6.

**Figure 7:**
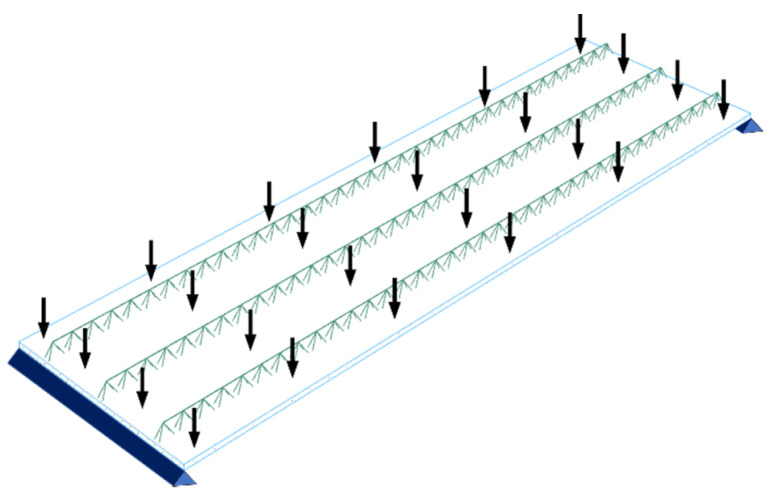
Loading and boundary conditions of Filigree ceiling slabs.

Boundary conditions in both 3D numerical models of a simply supported type were applied at two opposite shorter sides. The Filigree ceiling slabs with Type-D and Type-EQ trusses were loaded with a load evenly distributed over the entire surface of the plate (shown in Figure 7), which amounted to 150 kg/$m^{2}$ and 125 kg/$m^{2}$ for Type-D and Type-EQ trusses, respectively. Additionally, the reinforcement mesh and the bottom horizontal bars of the truss were anchored in the concrete slab using the embedded techniques available in Abaqus [36].

Two materials were used to describe the characteristics of individual elements in the examples analysed here: concrete and steel. The concrete was described by the linear stress–strain relations. For steel, an elastic perfectly-plastic model was used. The engineering parameters of materials used in the tests are shown in Table 1, where E is the Young’s modulus, ν is the Poisson′s ratio, and ρ is density.

In Model 1, the concrete cross-section was divided into 3D solid elements with dimensions of 30 mm in the thickness direction of the slab and 50 mm in the plane. This gives us 12,096 of 20-node brick elements with three degrees of freedom in each node (with reduced integration scheme, called C3D20R according to [36]). The steel components, i.e., truss and reinforcement mesh, were meshed using 2-node truss elements with three degrees of freedom (T3D2 according to [36]). Discretization gives a total of 963 and 1872 elements in the truss structure and reinforcement mesh, respectively.

Model 2, similarly to Model 1, was also discretized using 3D solid elements (C3D20R according to [36]) for concrete slab and truss elements (T3D2 according to [36]) for steel elements. The slab was divided into 7200 brick elements with the following dimensions: 25 mm in the thickness direction (to have at least two elements through the thickness) and 50 mm towards the plane of the slab. The total number of truss elements in the spatial truss structure was 807, and in the reinforcements mesh—1140 elements.

The total number of nodes in Model 1 equals 247,590 which gives in total 742,770 degrees of freedom (since all the elements have three degrees of freedom in each node). Model 2 consist of 147,894 nodes and thus has 443,682 degrees of freedom. These are rather sparse meshes, useful in this study, but if one would prefer to obtain more precise results, the number of nodes should be much higher, which in turn would cause much more demanding computations. In order to speed up computation but also to simplify the geometry for eventual variational study, a simpler model is required.

### 2.3. Representative Volume Element

**Figure 8:**
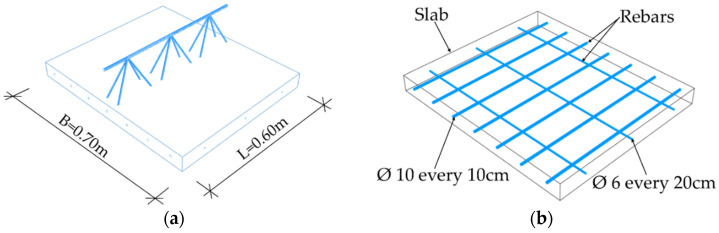

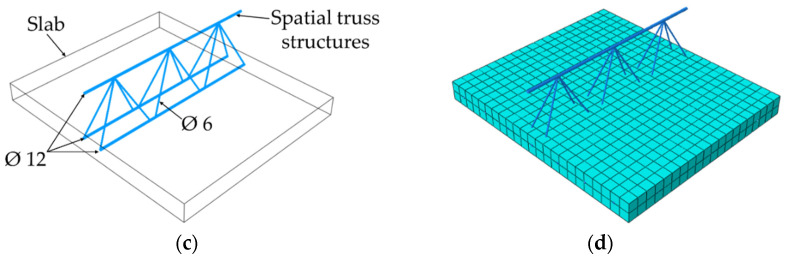
RVE-1—Type-D (**a**) geometry; (**b**) rebars; (**c**); spatial truss structure (**d**) FE mesh.

In order to perform numerical homogenization (details described further in the work), a proper definition of the stiffness matrix of a representative volume element (RVE) of a ceiling element is needed. RVE was created by extracting a periodic piece of structure from the entire 3D model. RVE for the Filigree ceiling slabs with D-type trusses was 0.70 m wide, 0.60 m long, and 0.06 m thick, see Figure 8a. For the correct definition of RVE, model geometry containing only one repetitive period is usually used. However, based on the experience gained, it was finally decided to use RVE with three periods. This is dictated by previous observations (see [21]), which show that for RVE with base dimensions deviating from the square, the transverse stiffness for the shorter side is overestimated. The choice of RVE with three periods has no effect on the remaining stiffnesses in the ABD matrix determined by this method. The slab was modelled as a solid 3D structure made of concrete. The model used repeatable D-type trusses (three periods) and reinforcement shown in Figure 8b,c. They were modelled as steel wire structures. In addition, the reinforcement mesh and the bottom bars of truss were embedded in the concrete slab. The slab was meshed with solid C3D20R elements, i.e., 20-node elements with three degrees of freedom, while the steel part was meshed with truss T3D2 elements (Figure 8d).

**Figure 9:**
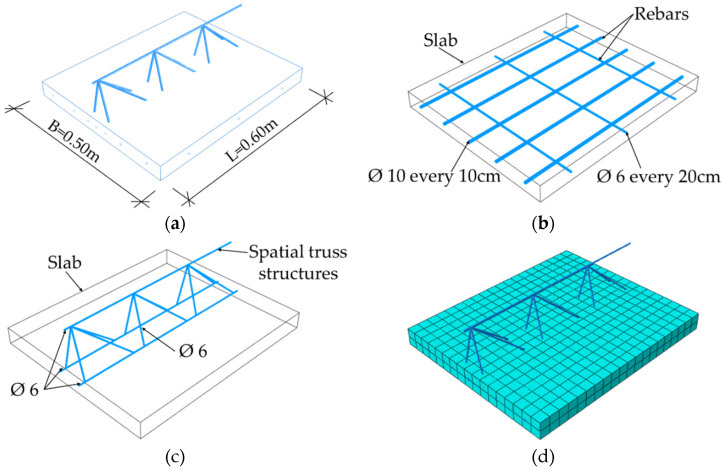
RVE 2—Type-EQ (**a**) geometry; (**b**) rebars; (**c**) spatial truss structure; (**d**) mesh.

The RVE model for the Filigree ceiling slab with EQ-type trusses consisted of a plate with dimensions of 0.50 × 0.60 × 0.05 m, EQ-type truss, and slab reinforcement, see Figure 9. The steel trusses and bars were modelled as a wire structure and then meshed with T3D2 type elements. The concrete part of the model was a 3D solid and was divided into FE using C3D20R elements.

### 2.4. Homogenization Algorithm

**Figure 10:**
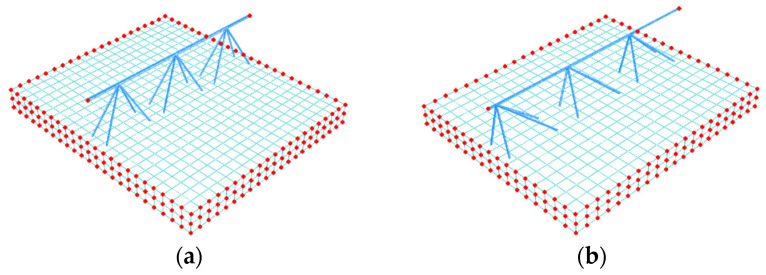
RVE—external (in red colour) and internal nodes (**a**) Type-D; (**b**) Type-EQ.

The classical formulation of a displacement-based linear finite element method includes

(1) $$K_{e}\;u_{e}=F_{e},$$

where $K_{e}$ is a statically condensed global stiffness matrix of the RVE; $u_{e}$ is a displacement vector of external nodes, and $F_{e}$ is a vector of the nodal force applied to external nodes. The FE mesh and external nodes are visualized in Figure 10.

Static condensation is the elimination of unknown degrees of freedom (DOF), also called secondary or minor degrees of freedom, here in relation to internal nodes, and re-formulation of the stiffness matrix with fewer unknowns reduced to the degrees of freedom in external nodes (referred to as the primary unknown or principal DOF).

The stiffness matrix condensed to external nodes (principal DOFs) can be calculated using the following equation:

(2) $$K_{e}=K_{ee}-K_{ei}\;K_{ii}^{-1}K_{ie}$$

where the global stiffness matrix is partitioned into external (subscript e) and internal (subscript i) nodes on four subarrays as follows:

(3) $$\left[\begin{array}{cc} K_{ee} & K_{ei}\\ K_{ie} & K_{ii} \end{array}\right]\left[\begin{array}{c} u_{e}\\ u_{i}\; \end{array}\right]=\left[\begin{array}{c} F_{e}\\ 0 \end{array}\right]$$

The total elastic strain energy stored in the system after static condensation is reduced to the work of external forces on the corresponding displacements and amounts to

(4) $$E=\frac{1}{2}u_{e}^{T}\;F_{e}$$

The energy equivalence between the simplified shell model and a full 3D finite element model can be presented by a proper definition of displacements at external nodes of RVE to trigger both membrane and bending behaviour. The generalized displacements at each node on the RVE edge surfaces are related to generalized strains. Originally proposed by Biancolini [20], later extended by Garbowski and Gajewski [21], the homogenization method was modified here to include only the translational degrees of freedom in both types of finite elements: truss and continuum. Therefore, the relationship between generalized constant strains and the position of external nodes on the RVE boundary is expressed by the following transformation:

(5) $$u_{i}=A_{i}\;\epsilon_{i},$$

where u is a displacement vector, ϵ is a strain vector. Here, for a single node ($x_{i}=x$, $y_{i}=y$, $z_{i}=z$), the $A_{i}$ matrix, adopted for a solid truss RVE model (after [20,21]), can be derived:

(6) $${\left[\begin{array}{c} u_{x}\\ u_{y}\\ u_{z} \end{array}\right]}_{i}=\left[\begin{array}{cccccccc} x & 0 & y/2 & z/2 & 0 & xz & 0 & yz/2\\ 0 & y & x/2 & 0 & z/2 & 0 & yz & xz/2\\ 0 & 0 & 0 & x/2 & y/2 & -x^{2}/2 & -y^{2}/2 & -xy/2 \end{array}\right]{\left[\begin{array}{c} \varepsilon_{x}\\ \varepsilon_{y}\\ \gamma_{xy}\\ \gamma_{xz}\\ \gamma_{yz}\\ \kappa_{x}\\ \kappa_{y}\\ \kappa_{xy} \end{array}\right]}_{i}.$$

The above matrix $A_{i}$ defines the relationship between displacements and effective strains, which are applied to the nodes on the boundary conditions (red points in Figure 10) to which the stiffness of the full model is condensed.

Recalling the definition of the elastic strain energy for a discrete model:

(7) $$E=\frac{1}{2}u_{e}^{T}\;K\;u_{e}=\frac{1}{2}\epsilon_{e}^{T}\;A_{e}^{T}\;K\;A_{e}\;\epsilon_{e}$$

and considering that for a shell (or plate), finite element subjected to bending, tension, and transverse shear, the elastic internal energy is

(8) $$E=\frac{1}{2}\epsilon_{e}^{T}\;A_{k}\;\epsilon_{e}\left\{area\right\}$$

thus, the stiffness matrix for a homogenized composite can be easily extracted from a discrete matrix as

(9) $$A_{k}=\frac{A_{e}^{T}\;K\;A_{e}}{area}.$$

The presented homogenization method adopted to truss-and-solid to shell transformation is based on the equivalence between the full 3D FE model and the simplified flat shell model by appropriate calculation of its effective stiffness. This allows for a significant acceleration of numerical analyses while maintaining a very high accuracy of the effective model.

In general, the $A_{k}$ matrix (which is the ABDR matrix) can be directly used in calculations by, e.g., general shell stiffness definition. However, if it is required to determine the effective material constants describing the orthotropic linear elasticity, first the effective thickness must be computed with the following equation:

(10) $$t=\sqrt{12\frac{trace\left(D^{*}\right)}{trace\left(A\right)}\;},$$

where

(11) $$D^{*}=D-BA^{-1}B,$$

Equation (11) can be easily suppressed by choosing the position of the neutral axis that minimizes the value of the B matrix. In such a case, $D^{*}=D$ The corresponding respective submatrices used in Equation (11) are

(12) $$D=\left[\begin{array}{ccc} D_{11} & D_{12} & 0\\ D_{12} & D_{22} & 0\\ 0 & 0 & D_{33} \end{array}\right];\;\;\;\;\;B=\left[\begin{array}{ccc} B_{11} & B_{12} & 0\\ B_{12} & B_{22} & 0\\ 0 & 0 & B_{33} \end{array}\right];\;\;\;\;\;A=\left[\begin{array}{ccc} A_{11} & A_{12} & 0\\ A_{12} & A_{22} & 0\\ 0 & 0 & A_{33} \end{array}\right].$$

In order to compute the effective parameters in the orthotropic model (represented by the four in plane material constants), the following decomposition of the D matrix (which describes bending stiffness) can be used:

(13) $$E_{11}^{D}=\frac{12}{t^{3}D_{11}^{-1}};\;\;\;\;\;E_{22}^{D}=\frac{12}{t^{3}D_{22}^{-1}};\;\;\;\;\;G_{12}^{D}=\frac{12}{t^{3}D_{33}^{-1}};\;\;\;\;\;\nu_{12}^{D}=\frac{D_{12}}{D_{22}}.$$

The same can be done with the A matrix (which describes the membrane stiffness):

(14) $$E_{11}^{A}=\frac{12}{tA_{11}^{-1}};\;\;\;\;\;E_{22}^{A}=\frac{12}{tA_{22}^{-1}};\;\;\;\;\;G_{12}^{A}=\frac{12}{tA_{33}^{-1}};\;\;\;\;\;\nu_{12}^{A}=\frac{A_{12}}{A_{22}}.$$

However, the most accurate results can be obtained by using average values (this is particularly important if the analysed model is expected to work in a complex membrane and bending state), which is calculated as follows:

(15) $$E_{11}=\frac{E_{11}^{D}+E_{11}^{A}}{2};\;\;\;\;\;E_{22}=\frac{E_{22}^{D}+E_{2}^{A}}{2};\;\;\;\;\;G_{12}=\frac{G_{12}^{D}+G_{12}^{A}}{2};\;\;\;\;\;\nu_{12}=\frac{\nu_{12}^{D}+\nu_{12}^{A}}{2}.$$

The effective transverse shear can be computed directly from the R matrix using the following equations:

(16) $$G_{13}=\frac{R_{11}}{t};\;\;\;\;\;G_{23}=\frac{R_{22}}{t},$$

where $R_{11}$ and $R_{22}$ are the transverse shear stiffnesses from the ABD matrix:

(17) $$R=\left[\begin{array}{cc} R_{11} & 0\\ 0 & R_{22} \end{array}\right].$$

## 3. Results

**Figure 11:**
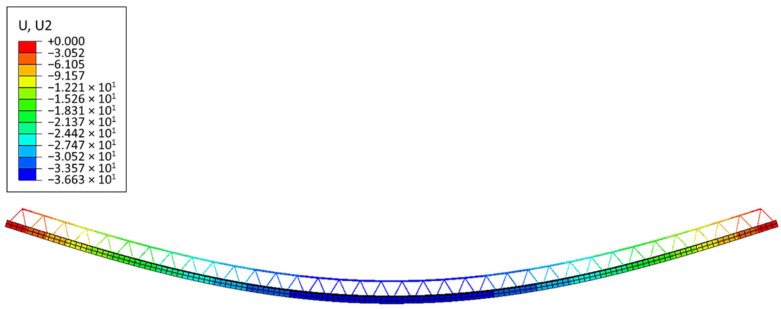
Vertical displacements of Model-1 (Type-D).

**Figure 12:**
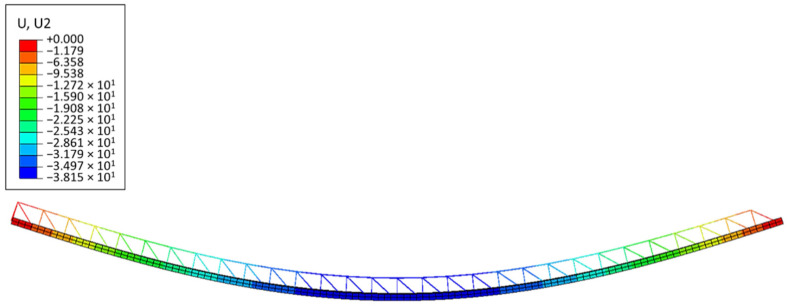
Vertical displacements of Model-2 (Type-EQ).

Below are the results of the numerical analysis presented for full 3D numerical models, Type-D (see Figure 11) and Type-EQ (see Figure 12). Figure 11 shows the side view of the slab loaded and displaced. The maximum displacement read from the numerical model equals 36.63 mm.

Figure 12 shows the vertical displacements of the numerical full 3D model of the slab (Type-EQ). The maximal deflection is in the centre of the plate and equals 38.15 mm. Both deflections are rather safe for the assembly of ceilings (in terms of failure); however, the development of models and their computation took significant time. If it is necessary to model a more complicated shape of the slab, the reconstruction of the full 3D geometry after each change seems unreasonable and it is certainly too time-consuming.

In contrast, the simplified single-layered, homogenized shell model of the slab is very easy to rebuild. In this model, the most demanding work is done prior to geometrical modelling, namely, during the homogenization process, which, however, is only done once for all. The stiffness matrix ABD, taking only the symmetrical part into account, is presented in Table 2. The table summarizes all three stiffness submatrices, namely, **A**, **B**, and **D** (shown in Equation (12)), as well as the transverse shear stiffness matrix **R** presented in Equation (17) for both RVE models. The model is referred to as the ABD Model hereafter in the results.

The effective thickness and all effective constitutive orthotropic constants computed from Equations (10)–(17) are presented in Table 3 for the RVE-1 model (Type-D), while the effective thickness and all effective material constants computed from Equations (10)–(17) for RVE-2 model (Type-EQ) are summarized in Table 4.

**Figure 13:**
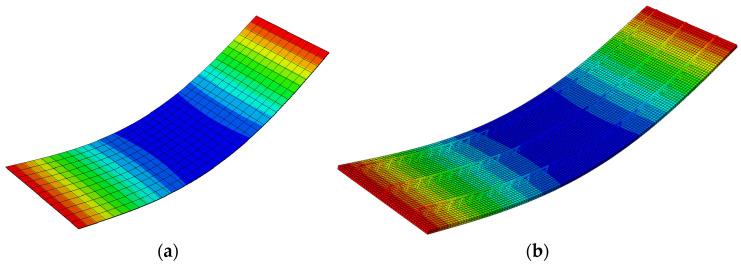
Comparison of vertical deflections: (**a**) RVE-1 shell model ABD; (**b**) full 3D FE model (Type-D).

A qualitative comparison of the deflected shape of the full 3D FE model and its simplified version (RVE-1, Type-D) is shown in Figure 13, while a quantitative comparison of the mid-span vertical deflection of the full 3D FE model and the corresponding simplified shell model (RVE-1, Type-D) is shown in Table 5.

**Figure 14:**
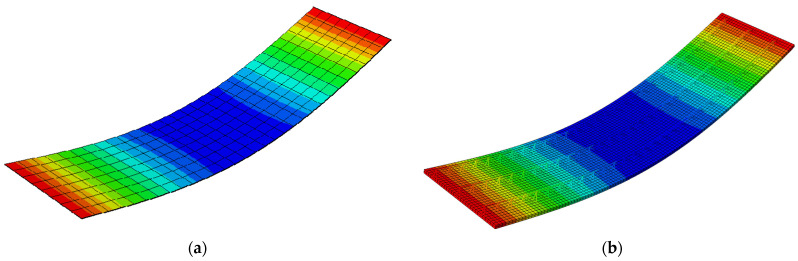
Comparison of vertical deflections: (**a**) RVE-2 shell model ABD; (**b**) full 3D FE model (Type-EQ).

Figure 14 shows a qualitative comparison of the deflected shape of the full 3D FE model of the Filigree slab (Type-EQ) and its simplified version (RVE-2). Table 6 summarizes the vertical displacement at the mid-span of the slab, calculated using the full 3D numerical model and the simplified model with three different cross-section definitions and material models (columns 3–5).

## 4. Discussion

The results presented in the previous section concern two models of Filigree type floor concrete prefabricated plates, namely, Type-D and Type-EQ, which differ in the thickness of the concrete slab and the geometry of the truss and the diameters of some bars. Both cases were analysed as simply supported plates with their own weight. In both cases, the maximum deflection did not exceed 40 mm.

The results obtained from the homogenization of relatively small periodic (repeatable) fragments of the plate, the so-called representative volumetric elements (RVE), are presented in Table 2. In both cases (RVE-1 and RVE-2), the membrane and bending stiffness in direction 2, which is parallel to the plate length, are much greater than in direction 1 (perpendicular to the L direction in plane). The ratio between the stiffnesses in the 2 and 1 direction for RVE-1 exceeded 3 and for RVE-2 was approximately 2.5. This is due to the peculiarities of the arrangement of spatial trusses that are oriented in the second direction. RVE-1 generally had much higher stiffness values than RVE-2, due to the slightly thicker concrete slab (60 mm vs. 50 mm), the lower truss structure, and greater main bar diameters (*ϕ* = 12 mm for RVE-1 and *ϕ* = 6 mm for RVE-2). It is worth noting that due to the coordinate system adopted in the homogenization process, the centre of which coincides with the centre of gravity of the concrete slab (in both analysed cases), the matrix **B** was practically zero.

The calculated material constants (summarized in Table 3 and Table 4) present an orthotropic elastic material. Orthotropy is the result of the homogenization process, which takes into account both the stiffness of the concrete slab (isotropic material) with the reinforcement in the form of a steel mesh and spatial steel trusses (again, isotropic material). The specific arrangement of the trusses and the number of rebars naturally generate direction-dependent behaviour for both compression/tension and bending/torsion stiffness, yet the orthotropy in both RVE-1 and RVE-2 was more apparent when the material parameters were calculated using the bending stiffness **D** than when calculating the membrane stiffness **A**.

The results (in Figure 13 and Figure 14 as well as in Table 5 and Table 6) show that virtually all three simplified models provided a precise displacement solution. This means that using the ABD stiffness directly (without defining the plate thickness or a constitutive model) in the numerical model as well as using models with the calculated material constants and effective thickness can be successfully used for quick engineering calculations. The main limitation of the proposed method, particularly when calculations use the ABD matrix directly, lies in the possibility of correct calculation restricted to displacements only. Models using the determined effective material parameters and effective thickness allow the analysis of stresses and strains as well; however, for an effective model, these will be effective values.

In further study, an extension to inelastic analysis of Filigree slabs is planned based on the general nonlinear constitutive law [37], already developed for 2D steel frames [38], composite beams [39], and corrugated sheets [40]. In this approach, plastic deformation can be captured by progressive degradation of stiffness.

## 5. Conclusions

The paper presents the numerical solid truss to shell homogenization method, based on the principle of strain equivalence. As a working example, an analysis of prefabricated floor slabs was carried out. The selected two types of filigree floor slabs differ in the spatial type of the truss. In order to verify the effectiveness of the proposed homogenization method, the full 3D models of the analysed slabs were built. Homogenized simplified models, both qualitatively and quantitatively, fully reflect the elastic behaviour of their full 3D FE models. The calculated displacements are more than in satisfactory agreement with the displacements calculated by solid truss FE numerical models. Thus, the method can be effectively used for quick and engineering calculations of composite plates with spatial trusses using simplified models, assuming that the correct homogenization of the selected plate was performed earlier.

## Figures and Tables

**Table 1:** Material parameters of steel and concrete used in the tests.

Material	E (GPa)	ν (−)	ρ $\left(kg/m^{3}\right)$
concrete	30	0.2	2400
steel	210	0.3	7900

**Table 2:** ABD stiffness matrix for RVE-1 and RVE-2 models.

Stiffness	RVE-1 Model ABD	RVE-2 Model ABD
A11 ($10^{5}$ MPa mm)	19.11	15.99
A12 ($10^{5}$ MPa mm)	3.83	3.19
A22 ($10^{5}$ MPa mm)	21.46	17.73
A33 ($10^{5}$ MPa mm)	7.50	6.25
B11 ($10^{5}$ MPa mm^2^)	0	0
B12 ($10^{5}$ MPa mm^2^)	0	0
B22 ($10^{5}$ MPa mm^2^)	56.93	20.07
B33 ($10^{5}$ MPa mm^2^)	0.04	0.04
D11 ($10^{5}$ MPa mm^3^)	5658.9	3281.1
D12 ($10^{5}$ MPa mm^3^)	1162.2	676.7
D22 ($10^{5}$ MPa mm^3^)	15,319.5	6828.7
D33 ($10^{5}$ MPa mm^3^)	2293.9	1349.7
R44 ($10^{5}$ MPa mm)	0.67	0.53
R55 ($10^{5}$ MPa mm)	0.69	0.59

**Table 3:** Material properties computed after homogenization of the RVE-1 model. Three different sets of parameters using Equations (13), (14) or (15).

Material Par.	Shell Model A	Shell Model D	Shell Model AD
t (mm)	75.97	75.97	75.97
E11 (MPa)	24,259	15,245	19,752
E12 (MPa)	27,234	40,849	34,042
$\nu_{12}$ (−)	0.18	0.08	0.13
$G_{12}$ (MPa)	9869	6278	8074
$G_{13}$ (MPa)	883	883	883
$G_{23}$ (MPa)	907	907	907

**Table 4:** Material properties computed after homogenization of the RVE-2 model. Three different sets of parameters using Equations (13), (14) or (15).

Material Par.	Shell Model A	Shell Model D	Shell Model AD
t (mm)	58.57	58.57	58.57
E11 (MPa)	26,315	19,175	22,745
E12 (MPa)	29,190	39,793	34,492
$\nu_{12}$ (−)	0.18	0.10	0.139
$G_{12}$ (MPa)	10,678	8060	9369
$G_{13}$ (MPa)	895	895	895
$G_{23}$ (MPa)	1003	1003	1003

**Table 5:** The deflection at the mid-span computed using the full 3D model (Type-D) and three simplified shell models (based on RVE-1 homogenization FE model).

	Full 3D FE Model	Shell Model ABD 1	Shell Model AD	Shell Model D
Deflection at the centre (mm)	36.63	35.70	42.97	36.13

^1^ This result were obtained by direct use of the ABD matrix.

**Table 6:** The deflection at the mid-span computed using the full 3D (Type-EQ) model and three simplified shell models (based on RVE-2 homogenization FE model).

	Full 3D FE Model	Shell Model ABD ^1^	Shell Model AD	Shell Model D
Deflection at the centre (mm)	38.15	38.32	44.24	38.37

^1^ This result were obtained by direct use of ABD matrix.

## Data Availability

The data presented in this study are available on request from the corresponding author.
